# Diagnostic Benefit of High b-Value Computed Diffusion-Weighted Imaging in Patients with Hepatic Metastasis

**DOI:** 10.3390/jcm10225289

**Published:** 2021-11-14

**Authors:** Maxime Ablefoni, Hans Surup, Constantin Ehrengut, Aaron Schindler, Daniel Seehofer, Timm Denecke, Hans-Jonas Meyer

**Affiliations:** 1 Department of Paediatric Radiology, University of Leipzig, Liebigstraße 20a, 04103 Leipzig, Germany; 2 Department of Diagnostic and Interventional Radiology, University of Leipzig, Liebigstraße 20, 04103 Leipzig, Germany; hans.surup@medizin.uni-leipzig.de (H.S.); constantin.ehrengut@medizin.uni-leipzig.de (C.E.); timm.denecke@medizin.uni-leipzig.de (T.D.); hans-jonas.meyer@medizin.uni-leipzig.de (H.-J.M.); 3 Department of Hepatology, University of Leipzig, Liebigstraße 20, 04103 Leipzig, Germany; aaron.schindler@medizin.uni-leipzig.de; 4 Department of Visceral, Transplantation, Vascular and Thoracic Surgery, University of Leipzig, Liebigstraße 20, 04103 Leipzig, Germany; daniel.seehofer@medizin.uni-leipzig.de

**Keywords:** computed diffusion-weighted imaging, high b-value, hepatic metastasis, MRI

## Abstract

Diffusion-weighted imaging (DWI) has rapidly become an essential tool for the detection of malignant liver lesions. The aim of this study was to investigate the usefulness of high b-value computed DWI (c-DWI) in comparison to standard DWI in patients with hepatic metastases. In total, 92 patients with histopathologic confirmed primary tumors with hepatic metastasis were retrospectively analyzed by two readers. DWI was obtained with b-values of 50, 400 and 800 or 1000 s/mm^2^ on a 1.5 T magnetic resonance imaging (MRI) scanner. C-DWI was calculated with a monoexponential model with high b-values of 1000, 2000, 3000, 4000 and 5000 s/mm^2^. All c-DWI images with high b-values were compared to the acquired DWI sequence at a b-value of 800 or 1000 s/mm^2^ in terms of volume, lesion detectability and image quality. In the group of a b-value of 800 from a b-value of 2000 s/mm^2^, hepatic lesion sizes were significantly smaller than on acquired DWI (metastases lesion sizes b = 800 vs. b 2000 s/mm^2^: mean 25 cm^3^ (range 10–60 cm^3^) vs. mean 17.5 cm^3^ (range 5–35 cm^3^), *p* < 0.01). In the second group at a high b-value of 1500 s/mm^2^, liver metastases were larger than on c-DWI at higher b-values (b = 1500 vs. b 2000 s/mm^2^, mean 10 cm^3^ (range 4–24 cm^3^) vs. mean 9 cm^3^ (range 5–19 cm^3^), *p* < 0.01). In both groups, there was a clear reduction in lesion detectability at b = 2000 s/mm^2^, with hepatic metastases being less visible compared to c-DWI images at b = 1500 s/mm^2^ in at least 80% of all patients. Image quality dropped significantly starting from c-DWI at b = 3000 s/mm^2^. In both groups, almost all high b-values images at b = 4000 s/mm^2^ and 5000 s/mm^2^ were not diagnostic due to poor image quality. High c-DWI b-values up to b = 1500 s/mm^2^ offer comparable detectability for hepatic metastases compared to standard DWI. Higher b-value images over 2000 s/mm^2^ lead to a noticeable reduction in imaging quality, which could hamper diagnosis.

## 1. Introduction

The most common malignant liver lesions are hepatic metastases, resulting in the most common indication for liver imaging [1]. As such, diagnosis with exact detection and localization of liver metastases are essential, as these can affect the clinical course of the malignant disease. Because of its high specificity and lack of radiation exposure, magnetic resonance imaging (MRI) has rapidly become the modality of choice for the detection and characterization of liver lesions [2,3]. The most sensitive MRI sequences for metastasis detection are diffusion-weighted imaging (DWI) and hepatobiliary phase (HP) images after intravenous application from a liver-specific contrast agent. Indicative features such as peripheral ring enhancement, diffusion restriction, and hypointensity on HP are typically seen in hepatic metastases [4,5,6].

Over the past decade, numerous studies have confirmed the diagnostic value of DWI in oncologic and non-oncologic applications [3,4,5,6,7,8,9]. The strength of DWI lies in its ability to qualitatively and quantitatively assess the diffusion properties of tissues and its ability to reflect tumor microstructure. In short, malignant tumors have higher cellularity, with resulting diffusion restriction detected by DWI [3,10]. Thus, DWI plays an essential role in tumor detection and characterization based on the high contrast between the lesion and surrounding tissue [11]. DWI increases the sensitivity and specificity for lesion detection in the liver [12,13]. However, DWI presents some limitations, such as low image resolution and poor signal-to-noise ratio (SNR), partly due to the short echo time (TE). Moreover, DWI can also be prone to image artifacts such as blurring or ghosting artifacts [5].

In hepatic diagnostics, DWI at higher b-values leads to low background signals from the normal liver parenchyma, which increases the contrast between the background liver and lesions. In clinical routine, b-values up to 800–1000 s/mm^2^ are acquired as high b-values [14]. There is still a lack of data for the diagnostic accuracy of high b-values over 1000 s/mm^2^ to date.

Lately, there has been a growing interest in the clinical assessment of computed DWI (c-DWI), especially in the field of oncology, with very promising results [8,14,15,16]. C-DWI is a mathematical postprocessing technique that produces virtually high b-values images by using real DWI data with at least two different lower b-values [17]. With this method, higher diffusion effects and SNR, by fitting input data with shorter TE, can be generated. Thus, the above-mentioned disadvantages of standard DWI could be avoided. Furthermore, c-DWI does not need additional acquisition time [17,18]. In other tumors, such as prostate cancer and breast cancer, higher b-value images showed higher conspicuity compared to standard b-value images [15,17].

For liver imaging, Shimizu et al. showed that there was no significant difference between acquired DWI and c-DWI images with b-values of 1000 s/mm^2^ in the detection of hepatic metastases at 3T [19].

Kawahara et al. validated the additional benefit of c-DWI with b-values of 1000 s/mm^2^ derived by a DWI obtained with lower b-values of 500 s/mm^2^ for the detection of hepatic metastases at 1.5T scanner [18].

One of the promising diagnostic abilities of c-DWI on higher b-values over 1000 s/mm^2^ is reduced T2 shine-through effect compared to standard DWI. This effect can lead to the misdiagnosis of benign, cystic lesions as malignant lesions due to the hyperintensity on the b 800 image. Another important aspect is that on higher b-value images, malignant lesions can have better conspicuity compared to standard DWI [5,15].

However, despite the promising diagnostic abilities of c-DWI, there is still no reliable data regarding the diagnostic value of c-DWI with higher b-values over 1000 s/mm^2^ for the diagnosis of hepatic metastases. This is of interest, as in other body localizations and tumor entities, a promising benefit of higher b-value DWI was proposed.

The aim of this study was therefore to investigate the usefulness of high b-value c-DWI in comparison to acquired DWI in patients with hepatic metastases and to compare different high b-values in regard to their visibility and extension of the liver lesions.

## 2. Materials and Methods

### 2.1. Patient Sample

This retrospective, single-center study was performed using a local hospital database to identify patients with known or suspected liver metastases, who received magnetic resonance imaging (MRI), including axial DWI and a gadoxetic acid-enhanced liver protocol with HP, between January 2018 and November 2020. All consecutive patients with histopathologic confirmed primary tumors with hepatic metastasis and available MRI were analyzed.

In total, 92 consecutive patients (39 females (42.4%)) with hepatic metastases were identified. The mean age was 60.8 years, and the range was 29–83 years (Table 1). The majority of primary tumor were colorectal carcinoma (*n* = 38, 41.3%), followed by malignant melanoma (*n* = 11, 12%), breast cancer (*n* = 7, 8%) and neuroendocrine tumors (*n* = 7, 8%). Out of all liver segments, Segments III and VII were the most affected (*n* = 54, 14.9% and *n* = 53, 14.6%, respectively).

### 2.2. Magnetic Resonance Imaging Studies

MRI was performed on a 1.5-T MR-scanner (Aera, Siemens Healthcare, Erlangen, Germany). All examinations were performed using a standard liver protocol, including unenhanced gradient echo (GRE) sequence, T2w turbo spin-echo (TSE) images, diffusion-weighted images (DWI) in axial orientation and fat-saturated T1w contrast-enhanced images, including the hepatobiliary late phase in the axial plane 20 min after contrast agent injection. All patients were administered a standard dose of 0.025 mmol/kg body weight gadoxetic acid (Gd-EOB-DTPA, Primovist/Eovist, Bayer HealthCare Pharmaceuticals, Berlin, Germany) as an intravenous injection at a flow rate of 1–2 mL/s, followed by a 20 mL saline flush. Axial DWI were either acquired with b-values of 50, 400 and 800 s/mm^2^ or 50, 400 and 1000 s/mm^2^. Over half of axial DWI were acquired with the highest b-value of 1000 s/mm^2^ (*n* = 52, 56.5%), and the other half were investigated with a b-value of 800 s/mm^2^ (*n* = 40, 43.5%). Computed higher b-values of 1000, 1500, 2000, 3000, 4000 and 5000 s/mm^2^ were generated using the “Philips IntelliSpace Portal” postprocessing software (Version 11; Philips, The Netherlands) with the “MR Advanced Diffusion Analysis”-application (Philips Health System, Hamburg, Germany). In short, the application employs a mathematical model that uses monoexponential equations to generate high b-value images. The protocol parameters are provided in Table 2.

### 2.3. Qualitative and Quantitative Analysis Image Analysis

Multiphase contrast-enhanced images were used as the gold standard to localize hepatic metastases, which were hypointense in HP. These lesions were then further analyzed with axial images of acquired and computed DWI.

**Figure 1:**
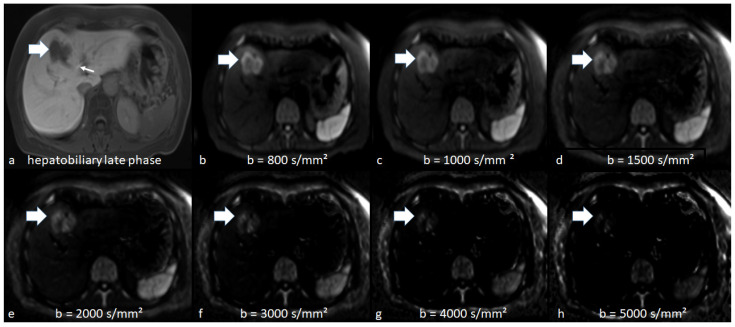
Illustrative patient with hepatic metastasis in Segment IVa/V (thick arrow) on hepatobiliary phase (HP) image (**a**), axial acquired (**b**) and computed diffusion-weighted imaging at high b-values of 1000–5000 s/mm^2^ (**c**–**h**). The thin arrow on HP indicates the biliary excretion of the contrast agent. On DWI at a b-value of 800 s/mm^2^ (**b**), the metastasis is equally visible than on c-DWI at b-values of 1000 (**c**) and 1500 s/mm^2^ (**d**) and becomes better visible compared than c-DWI images at higher b-values (2000–5000 s/mm^2^). At higher b-values (2000–5000 s/mm^2^ (**e**–**h**)), there is also a significant reduction in image quality.

For qualitative analysis, two readers (M.A. and H.S. with 3 years and 1 year of experience in general radiology, respectively) conducted a retrospective visual assessment independent of each other. A picture archiving and communication system (Syngo Plaza, Siemens Healthcare, Erlangen, Germany) was used. Each acquired DWI at b-values of 800 or 1000 s/mm^2^ and c-DWI with b-values of 1000, 1500, 2000, 3000, 4000 and 5000 s/mm^2^ were evaluated. The subjective detectability of hepatic metastases between different b-values was categorized as (0) worse delineation, (1) equal delineation and (2) better delineation compared to the adjacent surrounding tissue. Image quality was also assessed in regard to possible artifacts. The readers compared the b = 1000, b = 1500, b = 2000, b = 3000, b = 4000 and b = 5000 images simultaneously, side by side (Figure 1).

For quantitative analysis, the computed DWI images were compared with the acquired DWI of b = 800/1000 images in terms of lesion extension. Cases with discrepancies in the reviews were re-reviewed by both raters and discussed until a consensus was reached. Metastasis size defined as an area (cm^2^) was calculated on HP and DWI images separately by multiplying the manually measured maximal length and width of the lesion. The lesion volume was determined by multiplying the area with the amount of metastasis showing slices.

### 2.4. Statistical Analysis

All statistical analyses were performed using SPSS 24.0 (IBM SPSS Statistics for Windows, IBM Corp, Armonk, NY, USA). The Kolmogorov–Smirnov test was used to test the data of normal distribution. Categorical variables are presented as raw numbers and percentages. Normally distributed continuous variables are presented as mean ± standard deviation (SD) or median (interquartile range (IQR)) otherwise. Chi-square tests or the Mann–Whitney *U* test were used as appropriate to analyze descriptive data. The Mann–Whitney *U* test was used to compare lesion size on DWI. Interrater reliability regarding the detectability of hepatic metastases and image quality was assessed by intraclass coefficient (ICC) as follows: <0.20 = poor agreement; 0.21–0.40 = fair agreement; 0.41–0.60 = moderate agreement; 0.61–0.80 = good agreement; and 0.81–1.00 = excellent agreement. In all instances, a value of *p* = 0.05 was used for statistical significance.

## 3. Results

### 3.1. Quantitative Analysis

In the group in which c-DWI images were generated from DWI images at b-values of a maximum of 800 s/mm^2^ (*n* = 40, 43.5%), there were no statistically significant differences between the hepatic lesion sizes on DWI at b-values of 800 s/mm^2^ and on c-DWI at higher b-values of 1000 s/mm^2^ and 1500 s/mm^2^ (Table 3, e.g., *p* = 0.4 and *p* = 0.05, respectively). However, hepatic lesion sizes on DWI at b-values of 800 s/mm^2^ were significantly larger than hepatic lesions on c-DWI at b-values of 2000 s/mm^2^ and 3000 s/mm^2^. This was proven for both readers, with an interrater reliability ranging from 0.73 to 1.

In another group in which c-DWI images were generated from DWI images at b-values of maximum 1000 s/mm^2^ (*n* = 52, 56.5%), hepatic metastases on acquired DWI images were significantly larger than on c-DWI images at higher b-values (Table 3). Furthermore, at a high b-value of 1500 s/mm^2^, liver metastases were larger than lesions on c-DWI at b = 2000 s/mm^2^ (median: 10 cm^3^ (range: 4–24 cm^3^) vs. median: 9 cm^3^ (range: 5–19 cm^3^), *p* < 0.01) and b = 3000 s/mm^2^ (median: 10 cm^3^ (range: 4–24 cm^3^) vs. median: 5 cm^3^ (range: 2–14 cm^3^), *p* < 0.01). The interrater reliability ranged from 0.55 to 0.84.

In a subanalysis of colorectal carcinomas (b 800 group, *n* = 17, 18.5%; b 1000 group: *n* = 19, 20.5%) and another subanalysis of small metastases with a maximum transverse diameter of 1 cm (b 800 group: *n* = 12, 13.1%; b 1000 group: *n* = 20, 21.8%), hepatic metastases were always significant smaller in c-DWI at b = 2000 s/mm^2^ compared to acquired DWI (Table 4 and Table 5).

### 3.2. Qualitative Analysis

**Figure 2:**
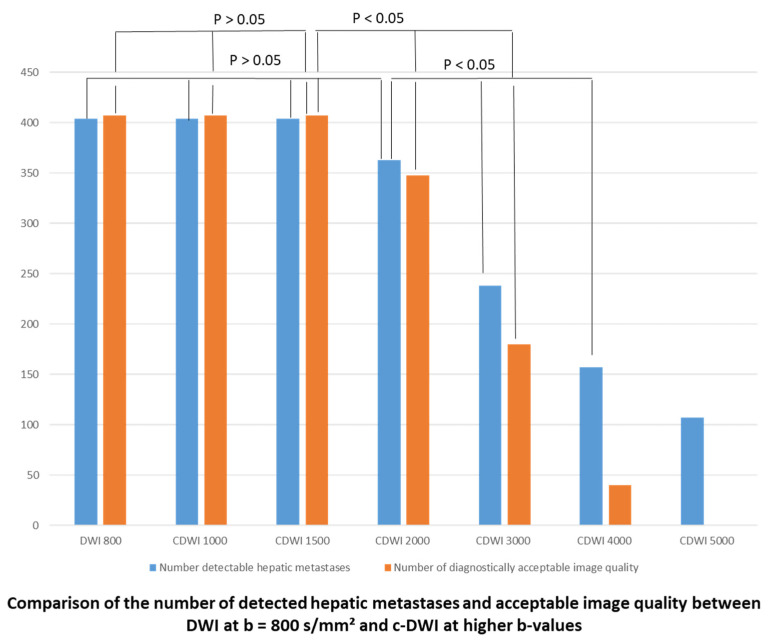
Bar graph displaying the number of detected liver metastases and acceptable image quality on DWI/c-DWI at b-values of 800–5000 s/mm^2^. There were no significant differences between b = 800 and b = 1000–2000 s/mm^2^ or between b = 800 and b = 1500 s/mm^2^ regarding detected metastases or image quality, respectively. However, the number of acceptable image quality dropped significantly from c-DWI at b = 1000 and c-DWI at b = 1500 s/mm^2^ to c-DWI at b = 2000–5000 s/mm^2^. There was also a significant reduction in the number of detected metastases from b = 2000 s/mm^2^ to b = 3000–5000 s/mm^2^.

Overall, in the b 800 group, 85% of all hepatic metastases were detected on DWI and c-DWI at b = 1000, 1500 and 2000 s/mm^2^ (Figure 2, *p* > 0.05). There was a statistically significant decrease in the number of detected metastases on c-DWI at a b-value of 3000 s/mm^2^ (b 2000 vs. b 3000 s/mm^2^: 34 (85%) vs. 17 (43%), *p* < 0.01).

Image quality dropped significantly from c-DWI images at b = 2000 s/mm^2^ to b = 3000 s/mm^2^ (number of images with diagnostically acceptable quality: b 2000 vs. b 3000 s/mm^2^: 34 (85%) vs. 15 (38%), *p* < 0.01). At b-values of 4000 s/mm^2^ and 5000 s/mm^2^, 99.7% of images were not diagnostic evaluable due to poor image quality (Figure 2). In 82% of all patients, the detectability of hepatic metastases was the same between DWI images and c-DWI images with high b-values ranging from 1000 to 1500 s/mm^2^. Starting from the high b-value of 2000 s/mm^2^, there was a clear reduction in image quality, with hepatic lesions being less visible compared to c-DWI images at b = 1500 s/mm^2^ in 80% of all patients. The interrater reliability ranged from 0.69 to 1.

**Figure 3:**
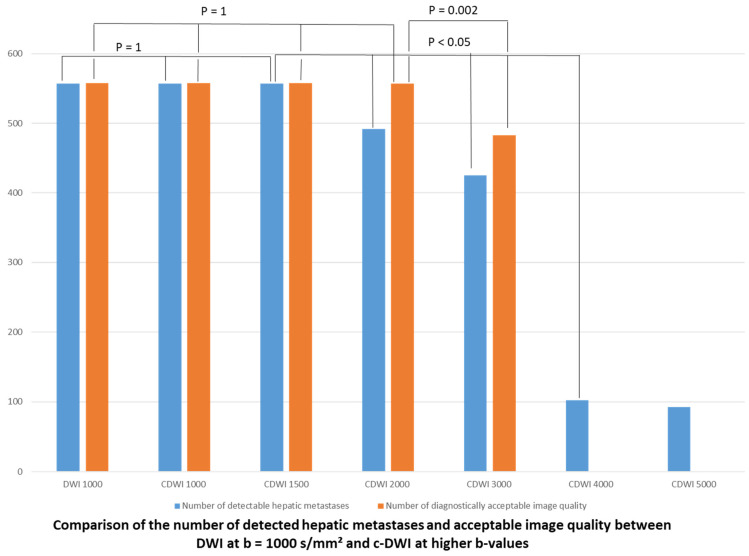
Bar graph displaying the number of detected liver metastases and acceptable image quality on DWI/c-DWI at b-values of 1000–5000 s/mm^2^. There were no significant differences between acquired DWI images at b = 1000 and c-DWI images at b = 1000–1500 s/mm^2^ or between b = 1000 and b = 1000–2000 s/mm^2^ regarding detected metastases or image quality, respectively. However, the number of acceptable image quality dropped significantly from c-DWI at b = 2000 s/mm^2^ to c-DWI at b = 3000 s/mm^2^. There was also a significant reduction in the number of detected metastases from b = 1500 s/mm^2^ to b = 2000–5000 s/mm^2^.

Finally, in the b-1000 group, at least 98% of all hepatic metastases were detected on DWI and c-DWI at b = 1000 and 1500 s/mm^2^ (*p* = 1). There was a statistically significant decrease in the number of detected metastases on c-DWI at a b-value of 2000 s/mm^2^ (Figure 3, b 1500 vs. b 2000 s/mm^2^: 51 (98%) vs. 42 (81%), *p* < 0.01). There was a significant reduction in image quality starting from c-DWI at b = 3000 s/mm^2^ (Figure 3 e.g., b 2000 vs. b 3000 s/mm^2^: 51 (98%) vs. 42 (81%), *p* < 0.01). There were no images of diagnostic quality on c-DWI at b = 4000–5000 s/mm^2^. In at least 82% of all patients, the detectability of hepatic metastases was the same between DWI images and c-DWI images with high b-values ranging from 1000 to 1500 s/mm^2^. Starting from the high b-value of 2000 s/mm^2^ there was a clear reduction in image quality with hepatic lesions being less visible compared to c-DWI images at b = 1500 s/mm^2^ in at least 82% of all patients. The interrater reliability was excellent, ranging from 0.82 to 1.

In a subanalysis of colorectal carcinomas and another subanalysis of small metastases with a maximum transverse diameter of 1 cm, there was no significant difference in the number of detected hepatic metastases between c-DWI up to b = 2000 s/mm^2^ and to acquired DWI (Table 6 and Table 7).

## 4. Discussion

The present study investigated the possible benefit of high b-values c-DWI in the diagnosis of hepatic metastasis. As shown, high b-values up to 1500 s/mm^2^ can potentially be used in clinical routine but do not provide a superior detectability compared to standard 800 and/or 1000 s/mm^2^ DWI images. As a second key finding, c-DWI images above 2000 s/mm^2^ cannot be recommended due to poor image quality.

Correct diagnosis of liver metastasis is very important for treatment planning in oncologic patients because even the diagnostic suspicion of a small liver metastasis can change the patient’s course from curative intended treatment to a palliative setting [1].

There is no doubt regarding the clinical benefit of DWI in liver imaging. It was shown that ADC values might be useful in the discrimination of benign to malignant liver lesions [20]. For liver metastasis in particular, DWI was identified to be very sensitive, even for small lesions below 1 cm in size [21]. It was clearly stated that metastasis detection with the addition of DWI and HP is superior to the conventional MRI technique, with a reported sensitivity of 0.88 in an analysis on colorectal liver metastasis of 1121 patients [22].

The definition of the best pair of b-values is of great clinical interest and can change the diagnostic abilities of DWI. In a study by Kaya et al., 124 hepatic lesions were evaluated, with 7 different b-values ranging from 0 to 1000 s/mm^2^ [4]. The authors concluded that b-values of 0 and 800 s/mm^2^ are preferable. In another meta-analysis of 1775 hepatic lesions, an overall pooled sensitivity of 0.86 and a specificity of 0.82 were reported for discrimination between malignant and benign liver lesions [23]. The authors further compared standard DWI obtained with b-values up to 1000 s/mm^2^ to low b-values in subanalyses, with significantly better accuracy for DWI based upon b-values of 800 and 1000 s/mm^2^. However, the authors concluded that there is a definite need for studies investigating the possible benefit of higher b-values [23]. A preliminary study on malignant primary liver tumors and metastases compared the diagnostic utility of different ADC values derived from b-values of 400, 800, 1600 and 2000 s/mm^2^ [24]. The authors could not identify significant differences of the different ADC values, but they did not assess the image quality of the high b-value images, as in the present study. In another interesting study, the addition of DWI to HP images significantly improved the diagnostic performance for residents [25]. In short, there is no doubt regarding the benefit of DWI in liver MRI. Yet, there are still uncertainties regarding the b-value choice.

There is a growing interest in c-DWI around oncologic imaging. The principal hypothesis is that the generated high b-values images allow a better lesion contrast with reduced T2 shine-through effect compared to standard DWI [17]. Due to the higher cellularity of malignant tumors, the diffusion restriction can be better visualized by high b-value images, as high b-value images are more sensitive to kurtosis effects [15,17]. As another important point, there is no need for further acquisition time.

For ischemic stroke imaging, there are reliable data that high b-value DWI can better display diffusion restriction, which was shown for acquired and computed images [6,7]. Notably, the b-value of 2000 s/mm^2^ had the best image quality [6,7].

For prostate cancer, a high b-value DWI of 1500 s/mm^2^ up to 2000 s/mm^2^ is recommended for the standard MRI protocol due to its superior diagnostic abilities compared to standard DWI. There are enough data that the computed high b-value image is as good as the acquired b-value image, as shown in a recent study employing b-values of 2000 s/mm^2^ [26]. There were even results that the c-DWI 2000 s/mm^2^ image might be superior in regard to image quality compared to the acquired one [27].

Similar results were reported for breast cancer patients [28]. The high c-DWI images up to 2000 in one study [29] and 2500 s/mm^2^ in another study [28] were of similar diagnostic quality compared to acquired images. For pancreatic cancer, it was recently published that c-DWI images with b-values of 1500 and 2000 s/mm^2^ are superior in visualization compared to standard DWI [30].

However, only one report was published regarding c-DWI in liver imaging. Kawahara et al. used a 1.5 T scanner and evaluated the diagnostic benefit of c-DWI b-values images of 1000 s/mm^2^ based on 56 patients with hepatic metastases [18]. The study could show that combined c-DWI and acquired DWI of 1000 s/mm^2^ is superior to acquired DWI alone. Yet, the authors did not evaluate c-DWI images with higher b-values.

Therefore, there are still no data regarding the possible benefit of high b-value DWI over 1000 s/mm^2^ for liver MRI. The present study provides new insight for c-DWI that contrary to other tumor entities, there is no diagnostic benefit of higher b-value images for liver metastasis based upon subjective measurements.

This might be caused by respiratory motion artifacts with possible misalignment of the acquired b-values, which could have an influence on c-DWI image quality [17]. Moreover, the influence of cardiac pulsation has a relevant effect, especially on high b-value images, which are sensitive to microscopic motion [17].

The present study measured tumor sizes on the different DWI images. Notably, metastases were smaller on c-DWI images of 2000 s/mm^2^ and above. This finding should be kept in mind when reporting the correct size of the metastases, and one should not use c-DWI images over 1500 s/mm^2^ for the measurement. There are several limitations of the present study to address. First, it is a retrospective study with possible inherent bias based upon a relatively small sample size. The reading was performed with the knowledge that liver metastasis is present, which could have an influence on the results. Second, due to the study design, we could not compare the c-DWI images with acquired high b-value images. It is therefore not known whether acquired high b-value images are superior compared to standard DWI and c-DWI images. Third, we could not perform subanalyses for primary tumors other than colorectal cancer due to the small sample size. Fourth, the present analysis was based on a subjective assessment of the DWI images. We did not assess quantitative parameters, such as SNR. Fifth, the volume assessment was performed as a columnar measurement, which is not as correct as a full volumetry. However, the performed measurement can easily be translated into clinical routine.

## 5. Conclusions

High c-DWI b-values up to b = 1500 s/mm^2^ offer comparable detectability for hepatic metastases compared to standard DWI acquired with b-values of 800 and 1000 s/mm^2^. Higher b-value-images over 2000 s/mm^2^ lead to a noticeable reduction in imaging quality, which severely reduces the diagnostic abilities.

## Figures and Tables

**Table 1:** Overview of the patient sample.

Characteristics	All Patients	
	*n*	%
	92	100
Gender		
Female	39	42.4
Male	53	57.6
Age	Mean: 60.8 years Range: 29–83 years	
Primary tumors		
Colorectal cancer	38	41.3
Malignant melanoma	11	12
Breast cancer	7	7.6
Neuroendocrine tumor	7	7.6
Pancreatic cancer	6	6.4
Lung cancer	4	4.2
Cholangiocarcinoma	3	3.3
Cervical cancer	3	3.3
Adenocarcinoma of the esophagogastric junction		
Gastric cancer	2	2.2
Gallbladder carcinoma	2	2.2
Renal cell cancer	2	2.2
Leiomyosarcoma	2	2.2
Urothelial carcinoma	2	2.2
Testicular cancer	1	1.1
Gastrointestinal stromal tumors	1	1.1
Hepatic metastasis	965	100 (100% of all patients)
Maximal transverse diameters <1 cm	291 (32 patients)	30.2 (34.8% of all patients)
Maximal transverse diameters ≥1 cm	674 (60 patients)	69.8 (65.2% of all patients)
Affected liver segments out of all 92 patients		
Segment I	25	6.9
Segment II	42	11.6
Segment III	41	11.3
Segment IV	49	13.5
Segment V	47	13.0
Segment VI	51	14.1
Segment VII	53	14.6
Segment VIII	54	15.0

**Table 2:** Sequence parameters of axial diffusion-weighted imaging (DWI) and hepatobiliary phase (HP) sequence.

1.5 T MRI Scanner		
Parameters	DWI (*n* = 93)	HP (*n* = 93)
FOV (mm × mm)	295 × 449	300 × 400
Matrix	134 × 88	320 × 180
ST (mm)	5	3
Number of Slices	114	72
TR (ms)	7900	3.56
TE (ms)	52	1.36
Flip angle (°)	90	10
b-values (s/mm^2^)	(*n* = 40, 43.5%) 50, 400 and 800	
	(*n* = 52, 56.5%) 50, 400 and 1000	

Abbreviations: FOV, field of view ST, slice thickness, TR, repetition time, TE, echo time.

**Table 3:** Lesion size comparison between acquired DWI images and computed DWI images or hepatobiliary phase sequence.

	HP	DWI	c-DWI b 1000	c-DWI b 1500	c-DWI b 2000	c-DWI b 3000	c-DWI b 4000	c-DWI b 5000
c-DWI derived from DWI b 800 images								
Volume: cm^3^ (IQR)	30 (6–50)	25 (10–60)	27.5 (10–56)	25 (10–50)	17.5 (5–35)	20 (5–34)	25 (15–25)	20 (14–20)
*p*-value (comparison with acquired DWI b 800 images)	0.82		0.4	0.05	0.001	0.001	0.5	0.5
c-DWI derived from DWI b-1000 images								
Volume: cm^3^ (IQR)	12 (6–29)	12 (5–31)	11 (5–25)	10 (4–24)	9 (4–19)	5 (2–14)	0	0
*p*-value (comparison with acquired DWI b 1000 images)	0.76		0.023	0.001	0.001	0.001		

**Table 4:** Lesion size comparison between acquired DWI images and computed DWI images or hepatobiliary phase (HP) sequence for hepatic metastases with colorectal carcinoma as the primary tumor.

	HP	DWI	c-DWI b 1000	c-DWI b 1500	c-DWI b 2000	c-DWI b 3000	c-DWI b 4000	c-DWI b 5000
c-DWI derived from DWI b 800 images								
Volume: cm^3^ (IQR)	39 (16–61)	30 (93–60)	37.5 (90–62)	30 (91–58)	30 (89–75)	25 (86–185)	0	0
*p*-value (comparison with acquired DWI b 800 images)	0.91		0.7	0.08	0.005	0.03		
c-DWI derived from DWI b 1000 images								
Volume: cm^3^ (IQR)	94 (9–111)	120 (10–113)	110 (10–90)	107 (8–85)	119 (8–92)	149 (3–108)	0	0
*p*-value (comparison with acquired DWI b 1000 images)	0.1		0.01	0.003	0.001	0.005		

**Table 5:** Lesion size comparison between acquired DWI images and computed DWI images or hepatobiliary phase (HP) sequence for hepatic metastases smaller than 1 cm.

	HP	DWI	c-DWI b 1000	c-DWI b 1500	c-DWI b 2000	c-DWI b 3000	c-DWI b 4000	c-DWI b 5000
c-DWI derived from DWI b 800 images								
Volume: cm^3^ (IQR)	4.6 (2.5–8)	4.8 (8–10)	4.8 (8–10)	4.6 (6.9–10)	3.4 (4.9–7.3)	1.9 (2.2–2.9)	not measurable	not measurable
*p*-value (comparison with acquired DWI b 800 images)	0.1		0.9	0.2	0.007	0		
c-DWI derived from DWI b 1000 images								
Volume: cm^3^ (IQR)	12 (6–29)	12 (5–31)	11 (5–25)	10 (4–24)	9 (4–19)	5 (2–14)	not measurable	not measurable
*p*-value (comparison with acquired DWI b 1000 images)	0.1		1	0.6	0.06	0.4		

**Table 6:** Comparison of number of detected small metastases under 1 cm diameter between acquired DWI images and computed DWI images.

	DWI	c-DWI b 1000	c-DWI b 1500	c-DWI b 2000	c-DWI b 3000	c-DWI b 4000	c-DWI b 5000
c-DWI derived from DWI b 800 images (*n* = 12)							
Number of detected metastases	106	106	106	106	78	30	30
*p*-value (comparison with acquired DWI b 800 images)		1	1	1	0.1	0.002	0.002
c-DWI derived from DWI b 1000 images							
Number of detected metastases	184	184	184	176	161	42	36
*p*-value (comparison with acquired DWI b 1000 images)		1	1	0.5	0.06	0	0

**Table 7:** Comparison of number of detected metastases from colorectal carcinoma between acquired DWI images and computed DWI images.

	DWI	c-DWI b 1000	c-DWI b 1500	c-DWI b 2000	c-DWI b 3000	c-DWI b 4000	c-DWI b 5000
c-DWI derived from DWI b 800 images (*n* = 12)							
Number of detected metastases	147	147	147	129	105	0	0
*p*-value (comparison with acquired DWI b 800 images)		1	1	0.5	0.008		
c-DWI derived from DWI b 1000 images							
Number of detected metastases	100	100	100	80	66	0	0
*p*-value (comparison with acquired DWI b 1000 images)		1	1	0.3	0.06		

## Data Availability

Qualified researchers may request access to patient level data and related study documents including the clinical study report, study protocol with any amendments, blank case report form, statistical analysis plan, and dataset specifications. Patient level data will be anonymized and study documents will be redacted to protect the privacy of trial participants.
